# A Tissue Renewal-Based Mechanism Drives Colon Tumorigenesis

**DOI:** 10.3390/cancers18010044

**Published:** 2025-12-23

**Authors:** Ryan M. Boman, Gilberto Schleiniger, Christopher Raymond, Juan Palazzo, Anne Shehab, Bruce M. Boman

**Affiliations:** 1 Department of Engineering, Drexel University, Philadelphia, PA 19104, USA; 2 Center for Applications of Mathematics in Medicine, Department of Mathematical Sciences, University of Delaware, Newark, DE 19716, USA; 3 Department of Mathematical Sciences, University of Delaware, Newark, DE 19716, USA; craymond@udel.edu; 4 Department of Pathology, Thomas Jefferson University, Philadelphia, PA 19107, USA; juan.palazzo@jefferson.edu; 5 CATX Inc., Princeton, NJ 08542, USA; ashehab@udel.edu; 6 Cawley Center for Translational Cancer Research, Helen F. Graham Cancer Center & Research Institute, Newark, DE 19713, USA; 7 Department of Pharmacology & Experimental Therapeutics, Thomas Jefferson University, Philadelphia, PA 19144, USA

**Keywords:** colorectal cancer, tissue renewal, systems biology, *APC* gene, familial adenomatous polyposis

## Abstract

Our Goal is to identify how colorectal cancer (CRC) arises in the single-layered cell epithelium (simple columnar epithelium) that lines the luminal surface of the large intestine. **Background:** We recently reported that the dynamic organization of cells in colonic epithelium is encoded by five biological rules and conjectured that colon tumorigenesis involves an autocatalytic tissue renewal reaction. Introduction Our objective was to define how altered crypt turnover explains tissue disorganization that leads to adenoma morphogenesis and CRC. *Hypothesis*: Changes in rate of tissue renewal-based cell polymerization leads to epithelial expansion and tissue disorganization during adenoma histogenesis. **Methods:** Accordingly, we created a computational model that considers the structure of colonic epithelium to be a polymer of cells and that tissue renewal is autocatalytic. Indeed, self-renewal of stem cells in colonic crypts continuously produces cells that act like monomers to form a polymer of cells (an interconnected, continuous cell sheet) in a polymerization-based process. Our model is a system of nonlinear differential equations that simulates changes in human crypt cell population dynamics. **Results:** We investigated how changes occur in the proportion of different cell types during adenoma development in FAP patients. The results show premalignant colonic crypts have a decreased rate of tissue renewal due to *APC*-mutation. **Discussion:** This slower rate of cell polymerization causes a rate-limiting step in the crypt renewal process that expands the proliferative cell population size. **Conclusions:** Our findings provide a mechanism that explains how a prolonged rate of crypt renewal leads to tissue disorganization with local epithelial expansion, infolding, and contortion during adenoma morphogenesis.:

## Simple Summary

Around 90% of colorectal cancer (CRC) tissues have driver *APC* mutations. Evidence that *APC* mutation drives tumor initiation and growth comes from familial adenomatous polyposis (FAP) patients who carry an *APC* germline mutation. If not surgically treated, FAP patients have a 100% risk for developing CRC. Our study of FAP patients evaluated changes in the dynamics of tissue renewal during early colon tumor development. The results show premalignant colonic crypts have a decreased rate of tissue renewal due to *APC* mutation. This slower rate of cell polymerization causes a rate-limiting step in the crypt renewal process that expands proliferative cell population size. This mechanism explains how a prolonged rate of crypt renewal causes tissue disorganization with local epithelial expansion, infolding, and contortion during adenoma morphogenesis. Since tissue renewal dynamically sustains cell numbers constant in all bodily organs, our findings also have significance in terms of understanding tumorigenesis in other cancer types.

## 1. Introduction

To identify mechanisms that explain how aberrant tissue renewal plays a role in colon tumorigenesis, we studied adenoma morphogenesis in familial adenomatous polyposis (FAP) patients. Indeed, tissue renewal determines the rate of cell division. In our quest, discovering why altered tissue kinetics occur was the crux of the matter. *Research Question*: How does dysregulation of the rate of colonic crypt renewal lead to the development of CRC? We recently reported that the dynamic organization of cells in colonic epithelium is encoded by five biological rules [1]. Our study showed that the organization of colonic tissue is encoded through temporal asymmetry of cell division whereby division of a mature parent cell produces two progeny cells with different temporal properties: a mature (cycling) cell and an immature (resting) cell. The precise timing, temporal order, and spatial direction of cell division provided a mechanism that explains how the organization of cells is dynamically maintained during tissue renewal. We surmised that these laws for the cellular organization of normal colonic epithelium might provide a means to understand how tissue disorganization occurs during cancer development. A clue came from another previous study on age-at-tumor diagnosis in FAP and sporadic CRC patients which indicated the mechanism for CRC development involves an autocatalytic polymerization reaction [2]. A reaction is autocatalytic if one of the reaction products is also a catalyst for the same reaction. In this view, the autocatalytic production of cells in tissue renewal may provide a mechanism that explains why colonic epithelium is self-sustaining. Hence, in our current study, we assumed that the colonic epithelium consists of a polymer of cells and investigated whether crypt renewal occurs via a cell polymerization process. Our study emerges from concepts on autocatalytic polymerization in engineering [3], mathematics [4], chemistry [5], and tumor biology [6]. In our thinking, autocatalytic cell polymerization is the mechanism that forms the epithelium which lines the colon and other hollow organs. To explore this mechanism, we modeled changes occurring in vivo during adenoma development to quantify the kinetics of crypt renewal in colon tumorigenesis. We conjecture the following: (1) Changes in the rate of crypt renewal correlate with phenotypic changes that occur during adenoma morphogenesis in FAP patients. (2) Altered kinetics of crypt renewal occur due to an autocatalytic polymerization-based mechanism in CRC development.

We selected hereditary CRC to investigate because CRC development progresses along the lines of normal colon to pre-malignant adenomas to CRC, and because histologic data on familial adenomatous polyposis (FAP) and sporadic patients is quantitative [7,8,9,10,11,12,13]. We have studied how the kinetics of dysregulated colonic crypt dynamics lead to stem cell overpopulation and colon tumorigenesis [14,15,16,17]. Indeed, it is widely accepted that CRC develops through an adenoma to carcinoma sequence in both FAP and sporadic CRCs [18,19] and that mutations in the adenomatous polyposis coli (*APC*) gene are the key driver mutational events in the initiation and development of most (>85%) CRCs [20].

Classic FAP patients develop 100s to 1000s of premalignant adenomas which demonstrates that *APC* mutations drive tumor growth in vivo. If FAP patients are left untreated, they have nearly a 100% risk of developing CRC. In FAP, germline *APC* mutations are the initiating event in adenoma morphogenesis, but loss of the second *wild type*-*APC* allele occurs during progression to full adenoma formation [21,22]. Two hits at the *APC* locus also occur as acquired mutations in the development of most sporadic CRCs [23]. While in both FAP and sporadic cases, the sequence of genetic and pathological events in CRC development are well-characterized [24], the kinetic mechanism that drives CRC initiation and progression is still not fully elucidated.

Herein, we took a three-pronged approach: (1) use microscopic analysis of normal, FAP, and adenomatous crypts to study the change in distribution of different cell populations during adenoma morphogenesis in FAP patients; (2) create a mathematical model for crypt renewal kinetics by simulating colon epithelium as a polymer of cells; and (3) apply the model to simulate quantitative pulse-labeling data on colonic crypt labeling indices. Then, we then determined if model output on tissue kinetic changes supports our hypothesis that changes in the rate of tissue renewal polymerization leads to epithelial expansion and tissue disorganization during adenoma histogenesis.

## 2. Methods

### 2.1. Microscopic Analysis of Ki67 Expression in FAP Colon Tissue Sections

Samples of normal human colon tissue were obtained from the distal margin of resection from individuals undergoing colon surgery, including, but not limited to, colon tumor resections. We investigated three types of tissues: (a) normal colonic crypts, (b) normal-appearing crypts from FAP patients, and (c) adenomatous crypts. The distribution of cycling cells in colonic tissues was illustrated by expression of the Ki67 marker for proliferating cells. Immunohistochemistry using 5 µm tissue sections was performed as we previously reported [25]. The primary antibody was anti-Ki-67, 1:100 (Immunotech, Westbrook, ME 04098, USA; Anti-Ki-67 mouse IgG1 Monoclonal Antibody, Unconjugated, Clone MIB-1, RRID:AB_86403). This study does not involve human participants in clinical trials, so randomization, inclusion and exclusion criteria, attrition, sex as a biological variable, age, weight, blinding, power analysis, replication, code information, data information, and protocol information were not part of the study. Formalin-fixed, paraffin-embedded sections of human colonic tissues were procured and obtained from our tissue procurement core facility. Patient consent was waived since patients were not recruited to this study as formalin-fixed, paraffin-embedded tissue blocks are considered secondary use and therefore consent was not required. The use of human tissues was approved by the Institutional Review Board of Christiana Care Health Services, Inc (FWA00006557; approval 6 April 2025). All tissue samples were de-identified and stripped of all direct identifiers so no information was available to identify the surgical patients from whom the tissue sections were derived. The patient tissue studies were conducted in accordance with the following ethical guidelines: Declaration of Helsinki, International Ethical Guidelines for Biomedical Research Involving Human Subjects (CIOMS), Belmont Report, and U.S. Common Rule.

### 2.2. Model Design for the Human Colonic Crypt

We created a mathematical model for the crypt by simulating colon epithelium as a polymer of cells. The model assumes that cell division produces cells that act as monomers that form polymers of cells (colon tissue) through an autocatalytic polymerization process. The model design contains a system of nonlinear differential equations that represent the changing dynamics of three cell populations (see below). The growth and death of all the cells is dictated by five equations.

(1) $$\mathrm{Symmetric}\;\mathrm{Division}\;C\;\overset{k_{1}}{\to }\;2C$$

(2) $$\mathrm{Autocatalytic}\;\mathrm{Polymerization}\;C+P\;\overset{k_{2}}{\to }\;2P$$

(3) $$\mathrm{Asymmetric}\;\mathrm{Division}\;P\;\overset{k_{3}}{\to }\;P+D$$

(4) $$\mathrm{Extrusion}\;D\;\overset{k_{4}}{\to }\;out$$

(5) $$\mathrm{Apoptosis}\;P\;\overset{k_{5}}{\to }\;out$$

In the model colonic crypt, there are three main cell types: *C* represents cycling (actively dividing) cells, *P* represents proliferative (non-cycling, G0-like, quiescent) cells, and *D* represents differentiated cells (unable to divide). The *P* and *D* cells make up the polymer of cells, with *P* making up the clonogenic portion of the crypt lining and *D* filling up the rest of the polymer lining. The *C* cells exist in the crypt lining that divides symmetrically, and daughter cells act like monomers that integrate into the polymer of cells through polymerization. Indeed, in colonic crypts, the biological self-renewal of stem cells continuously produces cells that act like monomers to form a polymer of cells (an interconnected, continuous cell sheet) in a polymerization-based process. Using the five equations above, the rate of growth of each type of cell can be determined.

(6) $$\frac{dC}{dt}=\left(k_{1}-k_{2}P\right)C$$

(7) $$\frac{dP}{dt}=\left(k_{2}C-k_{5}\right)P$$

(8) $$\frac{dD}{dt}=k_{3}P-k_{4}D$$

To be at equilibrium, the values must be

(9)–(11) $$C^{*}=\;\frac{k_{5}}{k_{2}},\;P^{*}=\;\frac{k_{1}}{k_{2}},\;D^{*}=\;\frac{k_{1}k_{3}}{k_{2}k_{4}}$$

The analysis of the system (Equations (6)–(8)) shows that this is a stable equilibrium and that a steady state is typically oscillatory as one would expect (see File S1).

## 3. Results

### 3.1. Staining for the Distribution of Cycling Cells in Normal and Neoplastic Colonic Crypts

**Figure 1:**
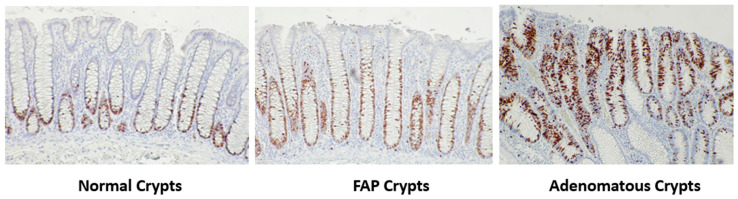
Ki67+ cell populations expand and shift upward along the crypt axis of FAP and adenomatous crypts. Illustration of how the distribution of cycling cells changes during colonic tumor development by expression of the Ki67 marker in colonic tissues. Note that showing changes in the distribution pattern of Ki67+ cells here is for qualitative illustration purposes only and was not quantified or included in our modeling analyses. Consequently, we immuno-stained colon tissue sections for Ki67 which is expressed during most active phases of the cell cycle (G1, S, G2, M) but is absent in early G1 and G0 phase cells [26]. Immunohistochemical staining for Ki67 expression was performed on normal colonic mucosa, normal-appearing colonic mucosa from FAP patients, and adenomas. In normal crypts, Ki67 staining shows that cycling cells are localized to the lower crypt but absent at the bottom-most region of the crypt located next to the muscularis mucosa. During the progression from normal colon to normal-appearing FAP epithelium to adenoma, these positively Ki67 staining cell populations expanded and shifted upward along the crypt axis.

To qualitatively illustrate how the distribution of cycling cells changes during colonic tumor development, we immuno-stained colon tissue sections for the expression of the Ki67 marker for proliferative cells. Note that Ki67 is expressed during most active phases of the cell cycle (G1, S, G2, M) but is absent in early G1 and G0 phase cells [26]. Figure 1 shows results from Ki67 staining in normal colonic mucosa, normal-appearing colonic mucosa from FAP patients, and adenomas. In normal, FAP, adenomatous crypts, Ki67 staining (Figure 1) shows cycling cells are localized to the lower crypt but absent at the bottom-most region of the crypt located next to the muscularis mucosa. During the progression from normal colon to normal-appearing FAP epithelium to adenoma, these positively Ki67 staining cell populations expanded and shifted upward along the crypt axis.

### 3.2. Quantitative Data on Labeling Indices

In our study, we used quantitative kinetic data derived from colonic crypt pulse-labeling indices (LIs) for model simulations (see Supplementary Materials S2). These data were selected because they were derived from measurements on the uptake of [^3^H] thymidine by cells in human colonic crypts which enables in vivo pulse labeling of DNA-synthesizing S phase cells [7,8,9,10,13]. Based on our analysis of these data, we were able to sub-classify crypt cells according to their temporal, kinetic phenotype and to quantitatively map their distribution along the crypt axis. The proportion (fraction) of S phase (labeled) cells when plotted against cell position (level) along the crypt axis shows proliferative shifts in FAP crypts (normal-appearing and adenomatous) [14,15]. Our further computations (see Supplementary Materials S2) give the distribution of (1) non-cycling differentiated (*D*) cells, (2) non-cycling, G0-like, proliferative (*P*) cells, and (3) cycling proliferative (*C*) cells in normal, FAP, and adenomatous crypts.

### 3.3. Model Output

We investigated the existence and stability of equilibrium solutions which were found analytically. The system has a first integral which shows that there is a neutrally stable equilibrium and that all other solutions oscillate around the equilibrium (Supplementary Materials S1). Using Matlab (version 2019b), the system was solved numerically. The numerical solution confirms the predicted behavior of the system.

**Figure 2:**
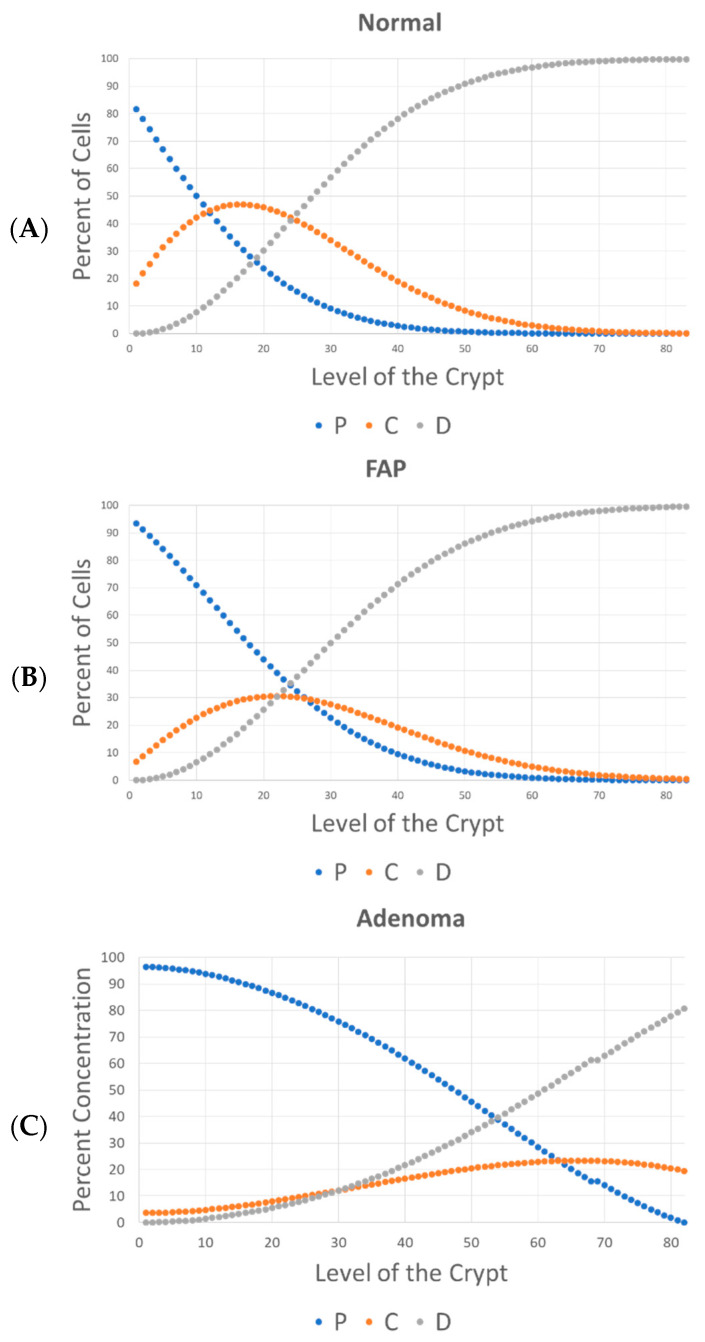
Indices of proliferative non-cycling (*P*), cycling (*C*), and differentiated (*D*) cell populations are righted-shifted in FAP and adenomatous crypts. These quantitative labeling indices show the percent of cycling (*C*), proliferative non-cycling (*P*), and differentiated (*D*) cells (*y*-axis) at each crypt level (*x*-axis; levels 1–82) for normal (**A**), FAP (**B**), and adenoma (**C**) crypts. In a colonic crypt, it can be determined that there are three main cell types: *P* represents proliferative (non-cycling) cells, *C* represents cycling (dividing) cells, and *D* represents differentiated cells (unable to divide). Data were derived as shown in File S2.

**Figure 3:**
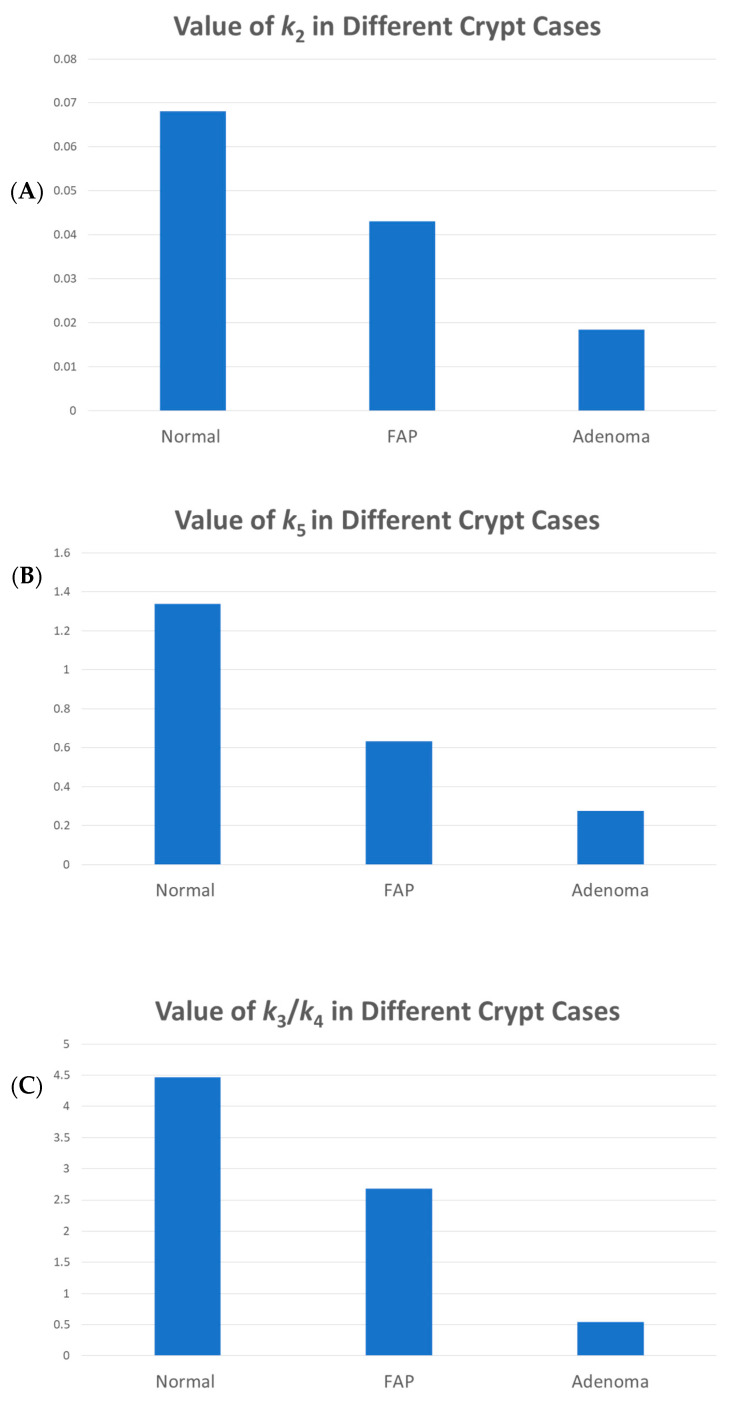
The rate of autocatalytic polymerization becomes increasingly slower during adenoma morphogenesis. Model output on rate constant values show the changes in rate constant values (*k*_2_–*k*_5_) between normal, FAP, and adenomatous tissues. Panel (**A**) (*k*_2_), Panel (**B**) (*k*_5_), Panel (**C**) (*k*_3_/*k*_4_). The progressive decrease in *k*_2_ and *k*_3_/*k*_4_ in normal-appearing and adenomatous crypts from FAP patients shows that the rate of autocatalytic polymerization (see Equations (2) and (3)) becomes increasingly slower during adenoma morphogenesis.

In equilibrium conditions, the percentage of each cell type can be expressed as a ratio of model rate constant values. To fit the parameters of the model, data on the percentage of *C*, *P*, and *D* cells in the colonic crypt (Figure 2) were used. Specifically, by measuring the area under the curve (AUC) of *C*, *P*, and *D* curves, shown in Figure 2, and by assuming that *k*_1_ is equal to one (i.e., time is measured in units of 1/*k*_1_), it is possible to determine the value of each *k* value at equilibrium based on Equations (9)–(11). Figure 3 shows the changes in rate constant values (*k*_2,3,4,5_) between normal, FAP, and adenomatous crypts. Results show that FAP and dysplastic (adenomatous) colonic tissues have decreased values of *k*_2_ (FAP crypts −1.6*X*-fold; adenomas −3.8*X*-fold), *k*_5_ (FAP crypts −2.6*X*-fold; adenomas −5.3*X*-fold), and *k*_3_/*k*_4_ (FAP crypts −1.6*X*-fold; adenomas −8.8*X*-fold) compared to normal tissues. The progressive decrease in *k*_2_ and *k*_3_/*k*_4_ in normal-appearing and adenomatous crypts from FAP patients shows that the rate of autocatalytic polymerization (see Equations (2) and (3)) becomes increasingly slower during adenoma morphogenesis.

**Figure 4:**
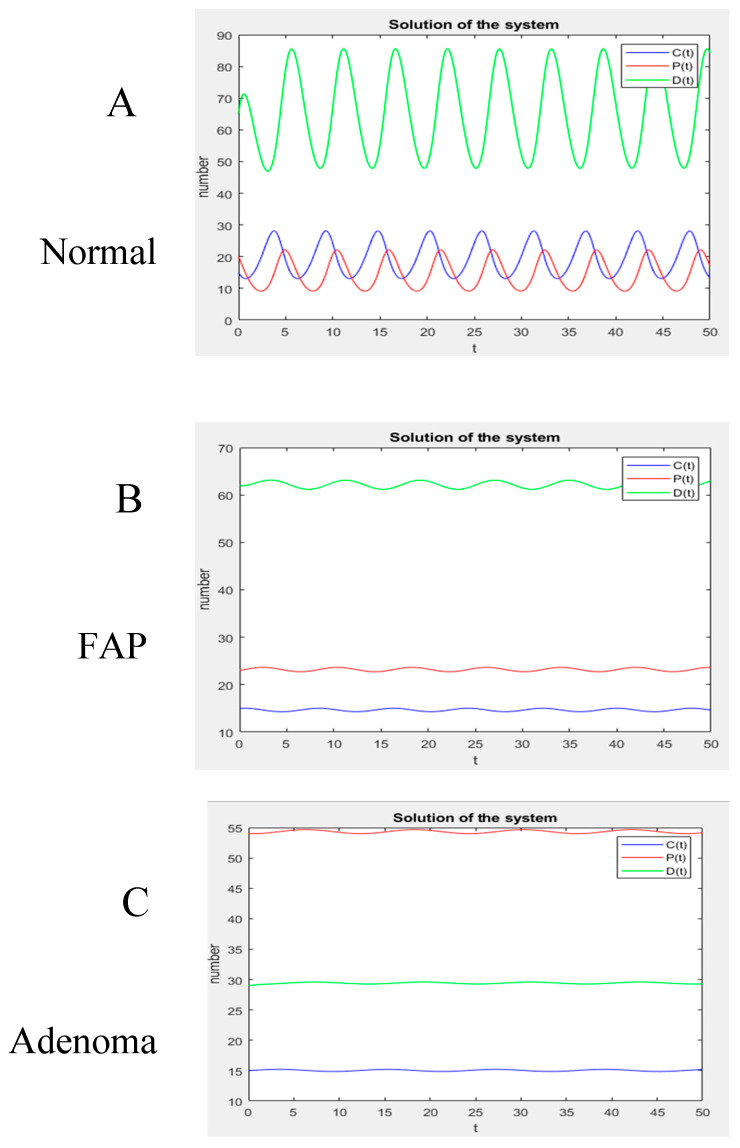
Non-cycling proliferative cells increase and differentiated cells decrease in *APC*-mutant crypts. Model output on colonic crypt kinetics shows that the model system will have an oscillating but stable steady state. These graphs show the number of *C* (cycling), *P* (proliferative non-cycling), and *D* (unable to divide) cells at each level of normal (**A**), FAP (**B**), and adenoma (**C**) crypts. This analysis shows that the number of non-cycling proliferative (*P*) cells drastically increases and the number of differentiated (*D*) cells decreases in *APC*-mutant crypts. The number of cycling (*C*) cells does not substantially change.

After the values of the rate constants were determined, a numerical computation was performed using Matlab, which reveals the dynamic behavior of the colon tissue systems. Notably, as shown in Figure 4, the model system will have an oscillating but stable steady state. Specifically, these plots (Figure 4A–C) show changes in the number of *C*, *P*, and *D* cells at each level of normal, FAP, and adenomatous crypts. According to this model, the increased number of *P* cells in FAP crypts (+1.4*X*-fold) and adenomatous crypts (+1.1*X*-fold) and the decreased number of *D* cells in FAP crypts (−1.1*X*-fold) and adenomatous crypts (−2.3*X*-fold) is due to changes in the rate constants (Figure 3). This finding indicates that the proportions of different cell populations progressively change in *APC*-mutant crypts (see Table 1), with a significant increase in the number of non-cycling proliferative cells and a decrease in the number of differentiated cells.

## 4. Discussion

The main finding in our study is that premalignant colonic crypts have a slower rate of cell polymerization, which leads to an increase in the percentage of proliferative cells and decrease in the percentage of differentiated cells in *APC*-mutant crypts. This discovery extends our previously reported findings that the dynamic organization of cells in colonic epithelium is encoded by five biological rules [1] and that colon tumorigenesis may involve an autocatalytic tissue polymerization reaction [2]. In comparison, the current study was designed to identify autocatalytic mechanisms at the tissue renewal level that lead to the development of colon cancer, which develops in colonic crypts that make up the intestine’s inner lining.

Accordingly, we created a mathematical model that considers the structure of colonic epithelium to be a polymer of cells and that colonic crypt renewal is autocatalytic. We then used the model to investigate changes in the proportion of different cell types occurring in adenoma development in familial adenomatous polyposis patients. Our analysis showed that a positive equilibrium exists that is neutrally stable. Given a steady state, it was possible to determine the value of each kinetic rate constant *k* value at each reaction step in the model. Results show that FAP and adenomatous colonic tissues have decreased rate constant values (*k*_2_, *k*_5_, *k*_3_/*k*_4_) compared to normal tissues, indicating that the rate of auto-polymerization is slower in premalignant crypts. This slower rate of cell polymerization causes a rate-limiting step in the crypt renewal process that expands the non-cycling proliferative cell population size. These findings support our *Hypothesis* that changes in the rate of autocatalytic cell polymerization in colonic crypts lead to a larger cell polymer in relation to epithelial expansion during adenoma histogenesis. Indeed, the histogenesis of adenomas involves local expansion and infolding of colonic epithelium [27,28]. This autocatalytic cell polymerization mechanism also explains how *APC*-mutation leads to a prolonged rate of crypt renewal that drives the early development of CRC.

**Figure 5:**
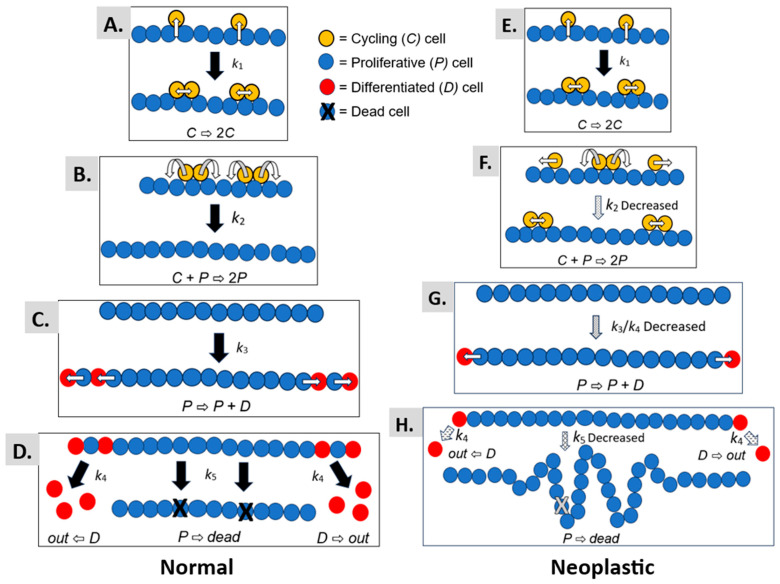
A slower rate of autocatalytic cell polymerization increases the non-cycling-proliferative cell population size and locally expands the colonic epithelium. The schema for autocatalytic polymerization kinetics shows the autocatalytic polymerization kinetics for normal (**A**–**D**) and neoplastic epithelium (**E**–**H**). The autocatalytic process for symmetric cell division occurs in both normal epithelium (**A**) and neoplastic epithelium (**E**) as a reaction with rate constant *k*_1_. The generation of new daughter cells and reinsertion into the single cell epithelial mono-layer based on the *k*_2_ rate constant simulates the polymerization process (**B**,**F**). The reaction with rate constant *k*_3_ models asymmetric cell division whereby a proliferative cell divides to produce a differentiated cell (**C**,**G**). Finally, a reaction with rate constant *k*_4_ for crypt cell extrusion and *k*_5_ for programmed cell death (apoptosis) is included in our model (**D**,**H**). Thus, based on changes in model kinetics (decreased *k*_2_ and *k*_3_/*k*_4_), a prolonged rate of colonic tissue renewal, enlarged non-cycling proliferative cell population, and local epithelial tissue expansion will lead to the infolding and buckling of colonic epithelium during adenoma histogenesis.

We provide a graphical illustration in Figure 5 to help understand the mechanism that explains how autocatalytic polymerization drives colorectal cancer development. The autocatalytic polymerization dynamics are illustrated for normal (Panels A–D) and neoplastic epithelium (Panels E–H). Several points below warrant further discussion on what our modeling results mean for the mechanism of CRC development.

### 4.1. Autocatalytic Polymerization Kinetics

The autocatalytic dynamic occurs because in the process of symmetric cell division, the cells self-renew, and through the elevator process, the polymer catalyzes its own production. In normal epithelia, symmetric cell division occurs through a process termed “elevator movement” [29,30]. In biology, the process of “elevator movement” is highly specific and precisely regulated. Indeed, it involves the coordination of many cellular mechanisms such as the orientation of mitosis in the intestine, retina, thyroid, and brain [31,32,33,34]. It begins with detachment of mitotic cells from the basal lamina in prophase, which allows cells to rotate. The process ends with reattachment of the daughter cells to the basal lamina in late metaphase. During elevator movement, mitotic cells round up and precisely orient their mitotic spindle, rotate, and divide in a specific orientation. Then, the daughter cells re-insert themselves into particular positions within the epithelium. Our model is designed to simulate the dynamics of this process as an autocatalytic reaction.

In our model, this process for symmetric cell division [35,36] is assumed to occur in both normal epithelium (Figure 5A) and neoplastic epithelium (Figure 5E) as a reaction with rate constant *k*_1_. The generation of a new daughter cell and reinsertion into the single cell epithelial monolayer simulates the polymerization process (Figure 5B,F). That is, insertion of a daughter cell into the epithelial sheet is like the process of building a polymer via combination of monomers together to form a polymer. This reaction model for cell polymerization is regulated by the *k*_2_ rate constant.

In our modeling, we found that the *k*_2_ rate constant is decreased in FAP and adenomatous epithelia (Figure 5F), which indicates that the rate of polymerization occurs more slowly in neoplastic epithelium. When the rate of polymerization is slowed down, it creates a rate-limiting step in the autocatalytic process. A slowed rate of autocatalytic polymerization could have two effects: (i) A prolongation of the time for symmetric cell divisions that occur before the polymerization reaction. An extended “induction” period might increase the number of mitotic cells. Indeed, increased mitotic figures (mitotic index) is a pathological hallmark of many tumors. (ii) A lengthening of the time for polymer growth. Extension of the time for polymer growth could lead to an increased cell polymer size and expanded epithelial sheet.

### 4.2. Cellular Differentiation Kinetics

Our model design also has a reaction with *k*_3_ rate constant for asymmetric cell division whereby a proliferative cell divides to produce a differentiated cell (Figure 5C,G). Additionally, the model has a reaction with *k*_4_ rate constant for the extrusion of differentiated cells from the polymer (Figure 5D,H). Our modeling of neoplastic epithelium shows that the *k*_3_/*k*_4_ ratio is decreased. In this case, a decreased *k*_3_/*k*_4_ ratio would cause an imbalance between asymmetric cell division and differentiated cell extrusion whereby the rate of asymmetric cell division becomes decreased relative to the rate of differentiated cell extrusion.

### 4.3. Cellular Apoptosis Kinetics

Finally, a reaction with *k*_5_ rate constant for programmed cell death (apoptosis) is also included in our model (Figure 5D,H). For neoplastic epithelium, our modeling revealed that the *k*_5_ rate constant is decreased, indicating that the rate of apoptosis is reduced (see Figure 5H). Taken together, a retarded rate of autocatalytic polymerization, a decreased ratio between asymmetric cell division and differentiated cell extrusion, and a decreased rate of apoptosis is predicted to cause local expansion of the epithelium that leads to infoldings of the single-layered cell epithelium. Collectively, the progression of these dysregulated reactions provides a basis for tumor histogenesis.

## 5. Conclusions

Our *Goal* was to identify how CRC arises in the single-layered cell epithelium (simple columnar epithelium) that lines the luminal surface of the large intestine. Accordingly, we created a mathematical model that considers the structure of normal colonic epithelium to be a polymer of cells. In our model, an autocatalytic polymerization-like mechanism is the basis of colonic tissue renewal. We then applied our model to understand how tissue changes occur in premalignant *APC*-mutant colonic epithelium during early human CRC development. In modeling neoplastic epithelium, our findings here show that a decrease in the rate of autocatalytic cell polymerization causes a rate-limiting step that leads to a larger polymer size. In this view, a slower rate of cell polymerization increases the non-cycling –proliferative cell population size and locally expands the colonic epithelium.

Thus, these findings support our *hypothesis* that changes in the rate of tissue renewal polymerization leads to epithelial expansion and tissue disorganization during adenoma histogenesis. Figure 5 illustrates how a prolonged rate of colonic tissue renewal, enlarged non-cycling proliferative cell population, and local epithelial tissue expansion leads to the infolding and buckling of colonic epithelium that occurs during adenoma histogenesis. Hence, our results provide a mechanism that explains how a tissue renewal-based polymerization mechanism drives colon tumorigenesis. Moreover, it is important to note that every tissue in the body has its specific tissue renewal time that controls the number and organization of cells in that organ [37,38]. Since tissue renewal is such a fundamental process in biology [39,40], the results from our study could significantly impact scientific understanding of how tumorigenesis occurs in the development of many cancer types.

## Figures and Tables

**Table 1:** The proportion of different cell populations is changed in *APC*-mutant crypts.

	*C* Cells	*P* Cells	*D* Cells
Normal	22%	17%	66%
FAP	14%	24%	62%
Adenoma	16%	54%	29%
FAP/normal	0.82	1.4	0.94 (1.1*X*-fold decrease)
Adenoma/normal	0.94	3.2	0.44 (2.3*X*-fold decrease)

## Data Availability

All data is available on request from the corresponding author (brboman@udel.edu). The code for modeling using MatLab software is found in Supplementary Materials S1. Data supporting reported results is found in Supplementary Materials S2 including any new data generated during the study.

## Supplementary Materials

The following supporting information can be downloaded at: https://www.mdpi.com/article/10.3390/cancers18010044/s1, File S1; File S2.
