# Supplementary material for: A Tissue Renewal-Based Mechanism Drives Colon Tumorigenesis
Source: Cancers (Basel). 2025 Dec 23;18(1):44. doi: 10.3390/cancers18010044 (PMC12784660; doi:10.3390/cancers18010044)

SUPPLEMENTAL MATERIAL File S1

Model design and analyses

$$\frac{dC}{dt} = (k_1 - k_2 P)C \tag{1}$$

$$\frac{dP}{dt} = (k_2 C - k_5)P \tag{2}$$

$$\frac{dD}{dt} = k_3 P - k_4 D \tag{3}$$

And its steady-states are $(C, P, D) = (0, 0, 0)$ and a positive steady-state

$$C^* = \frac{k_5}{k_2}, \quad P^* = \frac{k_1}{k_2}, \quad D^* = \frac{k_1 k_3}{k_2 k_4}. \tag{4}$$

Note that equation (3) decouples for (1),(2) so that it can be solved after the solution to (1)(3) is obtained.

To study the stability of the steady-states, we compute the eigenvalues of the Jacobian matrix of the system (1)-(3) at the steady-states.

$$J = \begin{pmatrix} k_1 - k_2 P & -k_2 C & 0 \\ k_2 P & k_2 C - k_5 & 0 \\ 0 & k_3 & -k_4 \end{pmatrix} \tag{5}$$

At the zero stead-state,

$$J = \begin{pmatrix} k_1 & 0 & 0 \\ 0 & -k_5 & 0 \\ 0 & k_3 & -k_4 \end{pmatrix}$$

has a positive eigenvalue, namely $\lambda_1 = k_1$. Hence, this steady-state is unstable.

At the positive steady-sate,

$$J = \begin{pmatrix} 0 & -k_5 & 0 \\ k_1 & 0 & 0 \\ 0 & k_3 & -k_4 \end{pmatrix}$$

has eigenvalues $\lambda_{1,2} = \pm i\sqrt{k_1 k_5}$ and $\lambda_3 = -k_4$. Hence stability is not determined by linear stability analysis, and a closer analysis of the system is needed. In particular, focusing on the system (1),(2) and perturbations off the steady-state, we define

$$C = \frac{k_5}{k_2}(1 + x) \quad \text{and} \quad P = \frac{k_1}{k_2}(1 + y), \tag{6}$$

where x and y measure deviations from the positive steady-state. If deviations do not grow, i.e. they either decrease or oscillate around zero, then the positive steady-state is stable.

Choosing a time-scale measured in terms of $1/k_1$, we define a dimensionless time $\tau = k_1 t$. Plugging into (1),(2), we obtain the reduced system

$$\frac{dx}{d\tau} = -(1+x)y \tag{7}$$

$$\frac{dy}{d\tau} = ax(1+y) \tag{8}$$

where $a = k_5/k_1 > 0$ is a parameter.

We can combine the equations to get

$$\frac{dy}{dx} = \frac{dy/d\tau}{dx/d\tau} = -\frac{ax(1+y)}{y(1+x)}$$

which is separable and can be integrated:

$$\int \frac{y}{1+y} dy = -a \int \frac{x}{1+x} dx$$

which yields

$$y - \ln(1+y) = -a\,(x - \ln(1+x)) + \beta$$

where β is a constant.

Since x and y are scaled versions of the perturbations in the first two variables, the fixed point corresponds to the solution $x = 0$, $y = 0$ to this system; this corresponds to $\beta = 0$. Note that this analysis could have been done in terms of the original variables as well.

Looking at the level curves of the function

$$y - \ln(1+y) + a\,(x - \ln(1+x))$$

for various values of $a > 0$, shows that they are closed curves around the fixed point (i.e. periodic solutions). The structure seems similar to the Lotka-Volterra predator-prey system: there are periodic solutions for this model, but the model is not structurally stable.

Conclusion: the positive steady-state is neutrally stable, i.e. solutions of (1),(2) corresponding to nonzero initial conditions oscillate around the steady-state.

To solve for D we define

$$D = \frac{k_1 k_3}{k_2 k_4}(1+z)$$

To obtain

$$\frac{dz}{d\tau} = b(y-z) \tag{9}$$

where $b = k_4/k_1 > 0$ is another parameter. Integrating (9), assuming $y(\tau)$ known, we obtain that

$$z(\tau) = z(0)e^{-b\tau} + b\int_0^\tau e^{-bs}y(\tau-s)ds \quad \approx \quad \int_0^\tau e^{-bs}y(\tau-s)ds \quad \text{for large } \tau\,,$$

And we conclude that z, and hence D will also oscillate with close to a fixed amplitude.

Note that the qualitative behavior of the system depends on two parameters, namely a the ratio of k_5 to k_1, and b the ratio of k_4 to k_1.

To solve (1)–(3) numerically using Matlab, we can solve the system (7)–(9) for $(x(\tau), y(\tau), z(\tau))$ and then recover

$$C(t) = \frac{k_5}{k_2}(1 + x(k_1 t)), \quad P(t) = \frac{k_1}{k_2}(1 + y(k_1 t)) \quad \text{and} \quad D(t) = \frac{k_1 k_3}{k_2 k_4}(1 + z(k_1 t)).$$

The solution is implemented in the script model.m that depends on odefun.m shown below.

1. **model.m**

```
% This script plots the solution to Ryan's model
%
% Propmpt for the user to provide relevant information
%
% Rate constants
k = zeros(5,1);
k(1) = input('k_1: ');
k(2) = input('k_2: ');
k(3) = input('k_3: ');
k(4) = input('k_4: ');
k(5) = input('k_5: ');
%
```

```
% Nondimensional time span
%
tf = input('Final time: ')
tauf = k(1)*tf;
tauspan = [0 tauf];
%
% Initial Conditions:
%
C0 = input('Initial condition C(0): ')
P0 = input('Initial condition P(0): ')
D0 = input('Initial condition D(0): ')
disp('C(0) = '); disp(' '); disp(C0);
disp('P(0) = '); disp(' '); disp(P0);
disp('D(0) = '); disp(' '); disp(D0);
%
% Times to plot solution
%
dtau = tauf/(20*round(tauf));
tau = [0:dtau:tauf];
%
% Define a, b and initial conditions
a = k(5)/k(1);
b = k(4)/k(1);
disp('a = '); disp(' '); disp(a);
disp('b = '); disp(' '); disp(b);
x0 = k(2)*C0/k(5)-1;
y0 = k(2)*P0/k(1)-1;
z0 = k(2)*k(4)*D0/(k(1)*k(3))-1;
w0 = [x0 y0 z0];
%
% Solve the system
%
[tau,w] = ode45(@(tau,w) odefun(tau,w,a,b), tauspan, w0);
t = tau/k(1);
y = w;
y(:,1) = k(5)*(1+w(:,1))/k(2);
y(:,2) = k(1)*(1+w(:,2))/k(2);
y(:,3) = k(1)*k(3)*(1+w(:,3))/(k(2)*k(4));
```

```
%
clf
% figure
plot(t,y(:,1),'-b','DisplayName','C(t)')
hold on
plot(t,y(:,2),'-r','DisplayName','P(t)')
plot(t,y(:,3),'-g','DisplayName','D(t)')
hold off
% plot(t,y(:,1),'-b',t,y(:,2),'-r',t,y(:,3),'-g')
xlabel('t')
ylabel('number')
title('Solution of the system')
lgd = legend;
```

2. **odefun.m**

```
function dwdt = odefun(t,w,a,b)
% Function giving the rates for C, P and D
% w is the vector (C,P,D).
dwdt = zeros(3,1);
dwdt(1) = -w(2).*(1+w(1));
dwdt(2) = a*w(1).*(1+w(2));
dwdt(3) = b*(w(2)-w(3));
end
```

Supplemental Materials File S2

The following new calculations on: 1) Non-cycling differentiated (D) cells, 2) Non-cycling, G_0-like proliferative (P) cells, and 3) Cycling proliferative (C) cells in normal, FAP, and adenomatous crypts provided new data that were used in the analyses performed in our current study.

In our previous studies of normal, FAP, and adenomatous colonic crypts [15, 16], we calculated the fraction of proliferative cells (F_{PR}), and the probability of a proliferative cell being in S phase (P_S). Specifically, the P_S and F_{PR} at each specific crypt level were derived using data from pulse-labeling of DNA-synthesizing S-phase cells. This data was based on uptake of [3H]-thymidine by cells in human colonic epithelium which provided a labeling index (LI) of crypt cell cycle kinetics [7-10, 13]. In our calculations, we assumed that the product P_S x F_{PR} computes the distribution of S-phase cells ($F_S = P_S$ x F_{PR}), where F_S corresponds to the biological LI. In our previous study [15, 16], we also derived cell cycle time (t_C) from the expression $t_C = t_S/P_S$, where t_S is the S phase time (8.8 hours) [11, 12]. These new calculations (sections 1-3 below) represent an extension of our previous studies, which provided data on proliferative cells that allowed us to determine the distribution of "D", "P", and "C" cells in human colonic crypts. Note, the population of proliferative (PR) cells consists of kinetically-distinct subpopulations: non-cycling, G_0-like (P) cells, and actively cycling (C) cells.

Below is the F_{PR} plotted as a percent of cells at each crypt level and the plot shows F_{PR} for normal, FAP, and adenomatous crypts as a function of crypt level [15, 16].

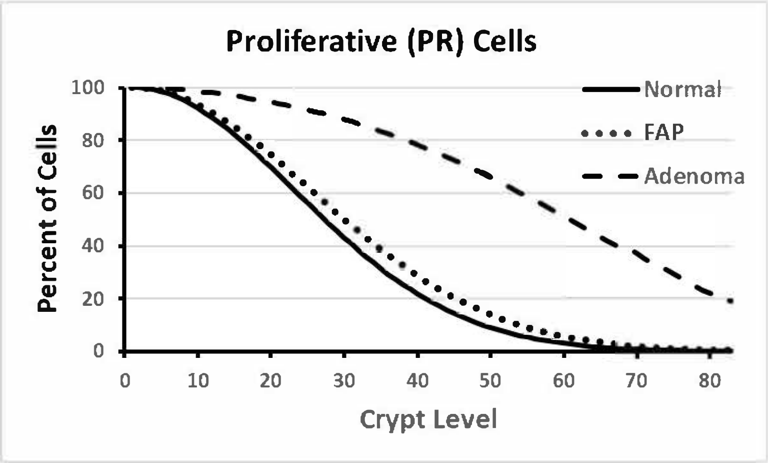

1. Calculations of non-cycling differentiated (D) cells in normal, FAP, and adenomatous crypts.

We next determined the F_D at each specific crypt level for normal, FAP, and adenomatous crypts. The F_D was calculated using F_{PR} (i.e. $F_D = 1 - F_{PR}$). The F_D graph below is plotted as a percent of cells at each crypt level and the plot shows F_D for normal, FAP, and adenomatous crypts.

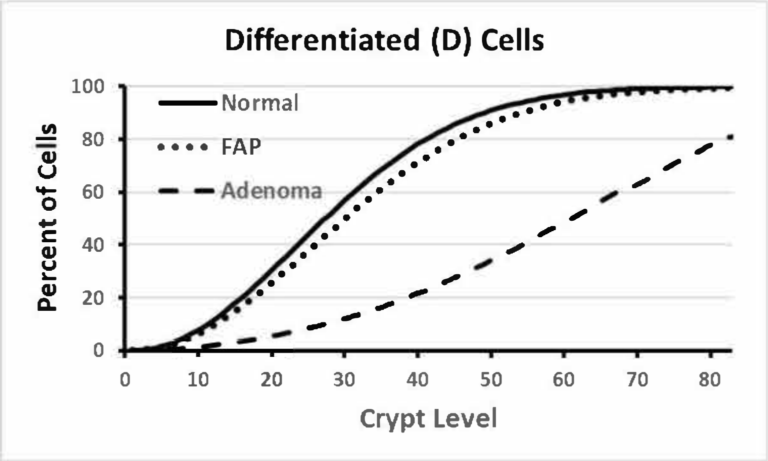

2. Calculations of non-cycling, G_0-like proliferative (P) cells in normal, FAP, and adenomatous crypts.

Additionally, we calculated the proportion of non-cycling proliferative (P) cells based on cell cycle time (t_C) as a function of crypt level in normal, FAP and adenomatous crypts. Determination of P cells was based on the following reasoning. The LI profiles indicate that there is a low fraction of cells in S-phase at the bottommost levels of the crypt. This is consistent with the fact that stem cell (SC) populations are quiescent, have a low frequency of being in S phase, and reside at the crypt bottom. Indeed, pulse-labeling for extended times of 2-3 days, which is beyond the average crypt cell cycle time of 24 hours (i.e. cycling cells are pulse-labeled within 1 day, labeling of non-cycling cells is >1 day) labels cells at the crypt bottom. This result shows that cells at the crypt bottom take extended time to incorporate [3H]-thymidine [7-10, 13]. Moreover, our previous study showed that the increase in non-cycling proliferative cells in FAP and adenomatous crypts is due an increase in a G_0-like, cell cycle latency period between end of M phase and beginning of G_1 phase [16]. Hence, our calculation of the probability of G_0 cells (P_{G0}) as a function of crypt level was done as follows. If P_{G0} is a function of cell cycle time (t_C), then $P_{G0} = t_{G0}/t_C$ and $t_{G0} = t_C - t_{G1\rightarrow M}$, which gives $P_{G0} = (t_C - t_{G1\rightarrow M})/t_C$. Based on the value for t_C (8.8 h) and $t_{G1\rightarrow M} = 15.92$ h [11, 12], the measurements for P_{G0} were obtained. Since $F_P = P_{G0} \times F_{PR}$, using our results on P_{G0} values and the F_{PR} values from Boman et al [15, 16], the fraction of F_P can be obtained. The F_P below is plotted for normal, FAP, and adenomatous crypts as a percent of cells at each crypt level.

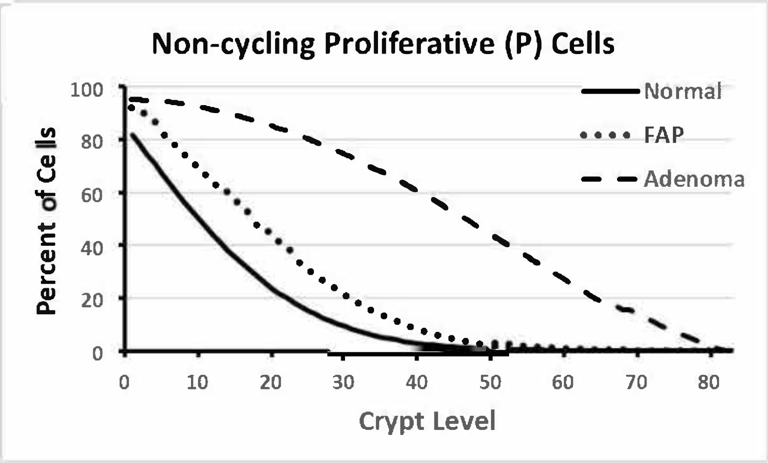

3. Calculations of cycling, proliferative (C) cells in normal, FAP, and adenomatous crypts.

We then calculated the distribution of cycling, proliferative (C) cells for FAP and adenomatous crypts. The fraction of F_C cells represents cycling cells in all phases of the cell cycle (G1→M). Thus, the F_C ($F_{G1\rightarrow M}$) was calculated based on the F_{PR} and F_P (i.e. $F_C = F_{PR} - F_P$). The F_C graph below is plotted for normal, FAP, and adenomatous crypts as a percent of cells at each crypt level.

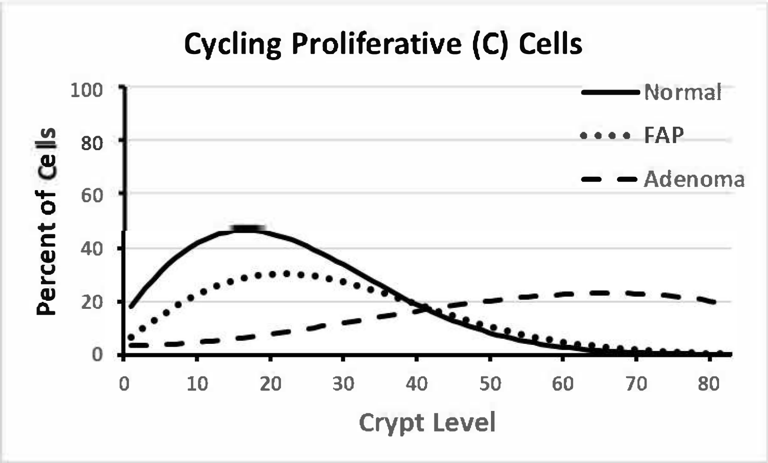

Supplement: Supplementary file 1 [file cancers-18-00044-s001.zip › cancers-3970931-supplementary.pdf]
